# Feeding and Swallowing Disorder in Adults With Intellectual Disabilities: Associated Factors

**DOI:** 10.1111/jar.70092

**Published:** 2025-07-17

**Authors:** Richella Kloppers, Dederieke A. M. Festen, Sandra Mergler

**Affiliations:** ^1^ Speech and Language Department ASVZ Care and Service Centre for People With Intellectual Disabilities Sliedrecht the Netherlands; ^2^ Department of General Practice and Intellectual Disability Medicine University Medical Centre, Erasmus MC Rotterdam the Netherlands; ^3^ Department of General Practice and Intellectual Disability Medicine Erasmus University Medical Center Rotterdam the Netherlands; ^4^ Medical Department ASVZ Care and Service Centre for People With Intellectual Disabilities Sliedrecht the Netherlands

**Keywords:** adults, dysphagia, feeding and swallowing disorder, health monitoring, intellectual disability, risk factor

## Abstract

**Background:**

Feeding and swallowing disorders (FSD)‐dysphagia are common in adults with intellectual disabilities and frequently overseen by caregivers.

**Aim:**

To determine the clinical factors that are relevant in daily practise associated with FSD in adults with intellectual disabilities.

**Method:**

Cross‐sectional data were collected from medical files. In addition, a digital questionnaire was sent to the primary caregiver. Logistic regression analysis was performed on beforehand determined and associated clinical variables.

**Results:**

In total, 106 participants (age 19 to 89) were included, and 54% of these participants were classified as having FSD. Variables positively associated with FSD were increasing mealtime support needs (*p* = 0.000), coughing (*p* = 0.004), cramming food (*p* = 0.027) and having severe‐profound intellectual disability (*p* = 0.001). The use of antipsychotic medication was negatively associated with FSD (*p* = 0.024).

**Conclusion:**

FSD is common in adults with intellectual disabilities and is associated with mealtime support needs, coughing, cramming food, the severity of intellectual disability, and the use of antipsychotic medication.

**Summary**

- FSD is common in adults with ID and is associated with mealtime support needs, coughing, cramming food, and having a severe to profound ID.
- FSD was found in 54% of the participants.
- It is implied to gain more attention to regular medical screening for factors associated with FSD.

## 1 Background

Feeding and swallowing disorders (FSD) are a common problem in adults with intellectual disabilities (Chadwick and Jolliffe 2009). In literature, different terminology is used to describe problems related to eating and swallowing. Dysphagia refers to disorders in the anatomic components of the oral, pharyngeal, or oesophageal stages of swallowing (American Speech‐Language‐Hearing Association 2019). The definition of FSD appears to be broader than dysphagia, as it includes the abnormal function in one or more phases of swallowing along with problems in eating activities that may be physiologic or psychological (Sheppard et al. 2014). FSD can have a big impact on the physical well‐being of adults with intellectual disability (Robertson et al. 2018). Dehydration, malnutrition, aspiration, airway blockage, and pneumonia are possible complications of FSD (Thacker et al. 2008; Perez et al. 2015). In severe cases, these complications can lead to death (O'Leary et al. 2018).

Besides the above‐mentioned physical effects, FSD can also have a negative effect on social and mental well‐being (Robertson et al. 2018). For example, fear of swallowing, aversion to drinks or food, and inability to dine with other people can be consequences of FSD.

Previous studies concerning the prevalence of FSD show varied outcomes in different subgroups of people with intellectual disabilities. International research showed that 8 to 11% of the adult population with intellectual disabilities has FSD (Robertson et al. 2017). However, in specific subgroups, the number of people with FSD appears to be higher. In the population ageing, people with intellectual disabilities a prevalence of 43.8% for swallowing difficulties was found (Sanders et al. 2024). Sheppard et al. (2014) reported a prevalence of 69.7% for mild to profound FSD among the group of adults and children with intellectual disabilities living in a residential setting. Differences in prevalence outcomes may also exist by different definitions of FSD that are used in different studies, for example, dysphagia by Robertson et al. (2017), swallowing difficulties by Sanders et al. (2024), swallowing and feeding disorder by Sheppard et al. (2014).

It is known that certain characteristics of people with intellectual disabilities are related to FSD. For example, it seemed to be more common in adults with moderate to severe intellectual disabilities (Robertson et al. 2017). Also, the presence of Down Syndrome, Cerebral Palsy, epilepsy, or dementia is associated with having a swallowing disorder (Perez et al. 2017; Robertson et al. 2017, 2018; Thacker et al. 2008). Additionally, previous studies showed that a history of respiratory infections in the previous year, increasing mealtime support needs, and being on psychotropic medication can be identified as associated factors of FSD (Evenhuis 2014; Perez et al. 2017; Robertson et al. 2018; Thacker et al. 2008).

FSD can be caused by neurological and anatomical dysfunction, or both (Arvedson 2008; Sheppard et al. 2014). In addition, some comorbidities make FSD a multifaceted problem (Sheppard et al. 2014).

As a consequence, diagnosing FSD can be challenging in people with intellectual disabilities. Due to cognitive and communication problems, FSD can be difficult to assess, and this may lead to a diagnostic delay (Robertson et al. 2018; Calis et al. 2008). Consequently, FSD may sometimes only be diagnosed after complications have occurred. However, early diagnosis is valuable to prevent negative effects on physical, mental, and social well‐being.

It is common practise that the detection of FSD in adults with intellectual disabilities mainly takes place by caregivers or relatives who are present during mealtimes. Therefore, the knowledge and alertness of the caregiver or family members concerning the symptoms of FSD are crucial for early detection. Unfortunately, previous research shows that FSD is frequently overseen by the caregivers (Calis et al. 2008; Trollor et al. 2017; Sanders et al. 2024).

Research on screening tools for FSD in people with intellectual disabilities is sparse (O'Leary et al. 2023).

Recently the screening instrument for Dysphagia in people with an Intellectual Disability (SD‐ID) was developed to assess the increased risk of FSD in adults with intellectual disabilities (van der Woude et al. 2023). This questionnaire, consisting of 29 items, is completed by the caregiver.

The Dutch “Signaleringslijst Verslikken” (SV) was developed as an applicable observational screening questionnaire by Helder (2010). The SV can be used by untrained caretakers to detect the presence of dysphagia in elderly people with intellectual disabilities. However, research showed that the SV is not suitable for detecting the presence of FSD in people with severe/profound intellectual and multiple disabilities aged 50 years and older, a group considered to be at high risk for having FSD (Van Timmeren et al. 2019).

The ‘Behavioral Assessment Scale of Oral Functions in Feeding’ developed by Ottenbacher et al. (1985), is another screening tool on FSD. However, this screening tool had limited generalizability as research took place only among those with severe/profound intellectual disabilities (O'Leary et al. 2023).

Finally, the Dysphagia Disorder Survey (DDS) is a frequently used tool to assess risk on FSD among adults with intellectual disabilities (Sheppard et al. 2014). The clinical application of this screening tool has been challenging as it is a standardised tool administered by trained specialists (O'Leary et al. 2023).

Overall, the current screening instruments are all questionnaires that have to be filled in by the caregiver or by a trained specialist. Besides, not all screening tools seem to be applicable for the heterogeneous group of adults with intellectual disabilities. In daily practise, care organisations for adults with intellectual disabilities include a heterogeneous group of people in which screening for FSD would be expedient. To prevent feeding and swallowing incidents, speech and language pathologists (SLP) should diagnose FSD in an early stage and not only after incidents have occurred. The current screening instruments are all instruments with questions that have to be filled in by the caregiver or the SLP.

Screening by caregivers for every person with intellectual disability connected to the care organisation is time‐consuming. Therefore, the group at risk for FSD should easily be identified for additional screening and possible diagnostics.

Despite the fact that some factors are most likely associated with FSD, a prediction model for clinical application on a heterogeneous group of adults with intellectual disabilities living in residential facilities has not been developed.

In the current study we focus on the risk inventory based on clinical factors related to FSD in order to develop a model for risk inventory based on information from the medical files. Thereby selecting a group at risk and in need of further screening and diagnosing FSD.

In practise, using such a model could release pressure on the caregiver and SLP. It will make the screening process more efficient and might reduce diagnostic delay and complications of FSD. Besides, it may increase insight at the risk of FSD in different groups in the organisation.

## 2 Aim

The aim of this study was to examine which factors that are relevant in daily practise are associated with FSD in adults with intellectual disabilities.

## 3 Methods

### 3.1 Design

This study presents the baseline data of a 2‐year follow‐up study designed for developing a validated multivariable prediction model in a heterogeneous group of adults with intellectual disabilities at risk for FSD.

### 3.2 Participants

Participants were recruited among the adult population with intellectual disabilities who received care from an intellectual disability physician and are connected to one care organisation. This organisation is sheltering a various population, including all levels of intellectual disability and all age groups. Inclusion criteria for participation were (1) being diagnosed with an intellectual disability, according to the formal diagnosis in the participants medical file, (2) age over 18 years, and (3) having an intellectual disability physician involved to provide medical care. Participants with a first registration within the past 2 years were excluded.

All 1633 adults meeting the eligibility criteria were included for random selection. Randomisation was performed using the random number generator at www.random.org. 453 eligible participants were selected and were informed about the study by their personal caretaker. They received an information letter with an invitation to participate in the study. Informed consent was obtained from all participants. For individuals who were incapable of giving their informed consent, approval was given by their legal representative.

Baseline data were collected between August 2020 and May 2021.

Registration of research data took place anonymously. A unique fictive code was used to trace back data by the first researcher.

The study has been conducted in full accordance with the World Medical Association Declaration of Helsinki. The study protocol was reviewed by the accredited medical ethics review committee of Erasmus MC Rotterdam (MEC‐2019‐0804). This study was found not to be subject to the Dutch Medical Research Involving Human Subjects Act.

### 3.3 Data Collection

The outcome variable of the model was FSD. In this study, FSD was defined as having at least one of the following criteria:

1. Officially diagnosed with a level 2,3,4,5 swallowing disorder on the Dysphagia Management Staging Scale (DMSS) in the medical record. Whereas level 1 is defined as no disorder, level 2 as mild disorder, level 3 as moderate disorder, level 4 as severe disorder, and level 5 as profound disorder (Sheppard et al. 2014). The DMSS is a clinical evaluation for rating the severity of eating and swallowing disorders based on management needs and health‐related outcomes. Adults with intellectual disability whose caretaker or relative suspected symptoms of FSD are screened with the DMSS, as usual care, by a speech and language pathologist (SLP).
2. A history of pneumonia by aspiration in the previous 2 years, officially diagnosed by a physician.
3. Classified as a mild, moderate, severe, or profound risk of dysphagia based on the caregivers' annual risk assessment. No risk noted or missing data in the annual assessment was classified as having no risk.
  The risk assessment contains a statement with a five‐level scale to rate the plausibility of aspiration and is part of the regular care process. This risk is established during the annual care plan discussion by the primary caregiver in collaboration with multiple care specialists and relatives.
4. A record in the registration system for choking incidents, in the previous 2 years.

Most data were collected retrospectively from medical files. The associated variables assessed were having Down syndrome, cerebral palsy, epilepsy, dementia, autism, gastroesophageal reflux or scoliosis, and use of antipsychotic or benzodiazepine medication. The level of intellectual disability was dichotomised into mild–moderate intellectual disabilities and severe–profound intellectual disabilities.

In addition to the use of medical files, a short digital questionnaire was sent to the primary caregiver by mail. This questionnaire consisted of 5 statements and assessed associated variables that were not reported in the medical record but were found to be relevant risk factors in the literature. Items were related to increasing mealtime support needs, increasing speed of eating, cramming food, premature loss of the food bolus and finally clearance of residuals from the upper airway during as well as after eating, for at least two times a week. Statements were answered by choosing yes or no. Data were collected directly after inclusion in the study.

### 3.4 Statistical Analysis

Data analysis was performed using IBM SPSS Statistics for Windows, version 22 (SPSS 2013; Armonk, New York., USA).

The rule of thumb was used to determine sample size. Ten events per variable were assumed. Besides, a prevalence of 50% was taken into account because no consensus is reached for the prevalence of swallowing difficulties in adults with intellectual disabilities.

Crosstabs *X* ^2^ were performed to analyse which variables were associated with the outcome FSD. Associated variables were analysed by logistic regression. A stepwise forward model building procedure has been done, using the Score test and significance defined as *p* < 0.05.

Parameters from the model were reported as odds ratio's (OR).

## 4 Results

Informed consent was obtained for 106 participants that were included in this study. Four participants opted out during the study. The reason for withdrawal was unknown. These cases were excluded from our study data. Table 1 presents the baseline characteristics of the 102 participants who took part in this study. In total, 55 participants (54%) were classified as having FSD. Of these, 60% were female. Mean age of this group was 53 years (range: 19–89, SD 14). Of the participants classified with FSD, 25 (46%) were officially diagnosed with the DMSS. Caregivers annual risk assessment classified 49 participants (89%) as having FSD, of whom 21 participants were also diagnosed with the DMSS. Twelve participants had a record in the registration system for choking incidents. For two participants, this choking incident recording was the only positive criterion.

**TABLE 1:** Participant characteristics.

		Swallowing difficulty yes (*n* = 55)	Swallowing difficulty no (*n* = 47)
Gender (female) *n* (%)		33 (60.0%)	23 (48.9%)
Age (mean; min–max)		53 (19–89 year)	48 (24–76 year)
Severity of intellectual disability. *n* (%)	Mild	5 (9.1%)	17 (36.2%)
	Moderate	16 (29.1%)	17 (36.2%)
	Severe	22 (40.0%)	10 (21.3%)
	Profound	12 (21.8%)	3 (6.4%)
Wheelchair dependent *n* (%)		14 (25.6%)	1 (2.1%)
Autism spectrum disorder *n* (%)		12 (21.8)	17 (36.2%)
Down syndrome *n* (%)		9 (16.4%)	5 (10.6%)
Epilepsy *n* (%)		18 (32.7%)	15 (31.9%)
Use of antipsychotics categorised by level of intellectual disability *n* (%)	Mild	3 (5.5%)	5 (10.6%)
	Moderate	4 (7.3%)	7 (14.9%)
	Severe	0	3 (6.4%)
	Profound	1 (1.8%)	1 (2.1%)

Crosstab analysis showed a significant association between six of the variables, for example, gastroesophageal reflux, level of intellectual disability, use of antipsychotics, increasing mealtime support needs, cramming food and coughing. The related value of each variable is reported in Table 2.

**TABLE 2:** Variables related to FSD based on crosstab analysis *X* ^2^.

Variable	Pearson *X* ^2^ value (df 1)	*p*
Antipsychotics	5.128	0.024*
Autism	2.388	0.122
Benzodiazepines	0.002	0.964
Cerebral Palsy	2.058	0.151
Coughing	8.358	0.004*
Cramming food	4.912	0.027*
Dementia^a^		0.059
Down syndrome	0.765	0.382
Epilepsy	0.023	0.880
Gastroesophageal reflux	7.309	0.007*
Mealtime support needs	14.784	0.000*
Premature loss of food bolus	3.141	0.76
Severity of intellectual disability	12.070	0.001*
Speed of eating	0.025	0.875

* Significant relationship: *p* < 0.05.

^a^ *X* ^2^ could not be performed; instead, a Fisher's exact test (two‐tailed) was used to determine if there was a significant association between dementia and swallowing disorder.

Increasing mealtime support needs was found to have the strongest association (*p* = 0.000). The above‐mentioned significant variables were conducted in a multiple forward stepwise logistic regression analysis (LR).

According to the model, the odds of having a profound‐severe intellectual disability for FSD is 1.023 times greater than the odds of having a mild–moderate intellectual disability. The log of the odds of adults with ID being on antipsychotics was negatively related to FSD. All odds are presented in Table 3.

**TABLE 3:** Logistic Regression Analysis of 101 adults with intellectual disabilities for swallowing difficulties.

Predictor	*β*	SE β	Wald's *Χ* ^2^	df	*p*	*e* ^β^ (odds ratio)	95% CI
Constant	−4.082						
Antipsychotics	−1.639	0.720	5.186	1	0.023	0.194	0.047; 0.796
Cramming food	1.203	0.544	4.895	1	0.027	3.331	1.147; 9.669
Support needs	3.113	1.112	7.842	1	0.005	22.499	2.546; 198.851
Coughing	1.422	0.647	4.825	1	0.028	4.145	1.165; 17.743
Severity of intellectual disability	1.023	0.516	3.939	1	0.047	2.783	1.013; 7.646
Test			*X* ^2^	df		*P*	
Overall model							
Likelihood ratio test			10.347	8		0.242	
Goodness‐of‐fit test							

In the logistic regression model, increasing mealtime support needs, coughing, cramming food, and having a profound‐severe intellectual disability were identified as positively associated variables after proven significance in linear analysis. The use of antipsychotic medication was negatively associated.

The multivariable model explained 45% of the variance (Nagelkerke *R* ^2^ = 0.457).

The goodness‐of‐fit statistic is the Hosmer–Lemeshow (H–L) test that yielded a *X* ^2^(8) of 10.347 and was insignificant (*p* > 0.05). Therefore, the null hypothesis of a good model fit to data was tenable.

## 5 Discussion

The present study explored the clinical and experimental factors found in the regular care process that are associated with FSD of adults with intellectual disabilities who received care from an intellectual disability physician and are connected to a care organisation.

In the logistic regression model, increasing mealtime support needs, coughing, cramming food, and having a profound–severe intellectual disability were identified as positively associated variables after proven significance in linear analysis. The use of antipsychotic medication was negatively associated.

Within this sample, which included men and women across the full range of age (19–89 years) and intellectual disabilities, 54% was classified as having FSD. It stands to reason that the prevalence of FSD would be directly affected by characteristics of the samples as well as diagnostic methods used (Robertson et al. 2017). Similar results were described by Hermans and Evenhuis (2014) and Sheppard et al. (2014) although research took place among different subgroups, respectively elderly and children with intellectual disability. The Dysphagia Disorder Survey (DDS) and a clinical dysphagia evaluation were used as diagnostic instruments in these studies. Van Timmeren et al. (2016) described a prevalence of 48% among adults with severe or profound intellectual disability and motor disabilities. In this study, dysphagia was diagnosed by having a registration in the medical record. The prevalence of FSD in the more heterogenous adult population with intellectual disabilities was 8%–11% (Robertson et al. 2017). Here, dysphagia had been confirmed by further investigations of an SLP. Compared to Robertson et al. (2017) the number of FSD found in the current study seems relatively high. However, in literature, different definitions are used to describe problems related to swallowing, for example, dysphagia, swallowing disorder, feeding disorder, and FSD. This probably explained the variation in prevalence found among different studies. A narrow definition like dysphagia will lead to a lower number, whereas a broader definition like FSD will assumedly lead to a higher number. For example, in the current study, the annual risk assessment of the caregivers and asphyxia incidents were also taken into account. If only the diagnosis by DMSS was used, the number of FSD would have been lower, namely 24.5%. However, this number is still higher than the 8%–11% found by Robertson et al. (2017). This difference may be explained by the generalizability of the sample. Robertson et al. (2017) described a sample that could be representative of the general population of adults with intellectual disability. In the current study, adults with intellectual disability living in a residential facility were selected. Perhaps more attention for FSD is embedded in residential care than it is in the general population, leading to higher prevalence rates. Also, people in residential care may be more affected by their disability and therefore have higher prevalence rates of FSD.

The use of antipsychotics was found to be negatively related to FSD, which was an unexpected result. Previous research done among different study populations found a positive effect of the use of antipsychotic drugs on pneumonia (Luykx et al. 2024; Knol et al. 2008; Gau et al. 2010; Trifiro et al. 2010). In the field of care for people with intellectual disability, we found one other study with a negative association between the use of antipsychotics and FSD (van der Woude et al. 2023). At present, it is unclear if the dosage of antipsychotic medication varied between different studies and study populations. Higher dosages might increase the risk of FSD in certain populations.

The odds of needing mealtime support were found to be relatively high (3.113) compared to the odds of cramming food, coughing, use of antipsychotic medication and severity of intellectual disability (respectively 1.203, 1.422, −1.639, 1.023). These findings highlight the importance of caregiver training on how to support people having FSD during mealtime. Previous research of Manduchi et al. (2021) showed that 35% of the adults with intellectual disability and receiving help with eating had a choking history.

In order to interpret the findings of this study, some limitations need to be considered. First, retrospective file data were used in the model construction. Therefore, there is a risk of missing data in medical files concerning the study variables. Besides, selection bias may have occurred as participants had to give their consent to participate in this study. For example, consent might have been given more often in participants known with feeding and swallowing problems.

Second, the lack of using a valid diagnostic instrument to confirm FSD affected the outcome. Retrospective data depend on the diagnoses reported in the medical file. In only 25% of the participants, FSD was officially confirmed by DMSS. From previous research, it is known that those with a confirmed diagnosis of dysphagia may only include those with more severe FSD (Chadwick and Jolliffe 2009). Besides, it is known that frequently no reference to swallowing difficulties is found in the medical record (Sanders et al. 2024). Therefore, only using a registration in the medical record as an outcome measure could lead to an underestimation of swallowing difficulties. In this study, a broad nature of outcome measures was used to include persons that otherwise may be undiagnosed if only DMSS as a registration in the medical record was used. The addition of these outcome measures showed a higher indication of FSD. Fifty‐one percent of the total FSD diagnoses was confirmed by the caregivers annual risk assessment. On the other hand, the validity and reliability of these added outcome measures have not been assessed. Van Timmeren et al. (2019) showed that an observational screening questionnaire (SV) filled in by caregivers for detecting the presence of FSD demonstrated inaccuracy in the range of mild to moderate dysphagia. Signs of milder dysphagia are often missed. The reasoning behind this is that health problems with less visible signs and symptoms will be easily overlooked by caregivers. Therefore, in this study, we paid attention to different outcome measures of FSD to include those who otherwise probably would be missed. In further prospective research, each participant should be diagnosed by a speech and language pathologist to verify the FSD outcome and validate the associated variables. Only by doing so will it be possible to obtain a realistic estimation of FSD.

Third, the group of participants is relatively small. In total 101 people participated in this study. It is considered possible that more significant results might be found in the case of a larger study population.

The main strength of this study is the random selection method used, including a heterogeneous study population of people with intellectual disability receiving formal support and not only subpopulations like elderly or people with severe intellectual disability. The study focussed on the population known to services instead of a disease specific classification. However, the effect of a possible selection bias is unknown. This may negatively affect the generalisability of the results.

This study presented baseline data as part of a longer cohort‐study developing a prediction model based on clinical and experimental factors found in the regular care process. This model will be developed to assess the risk on FSD, based on factors known from medical files. Currently, the associated factor ‘increasing mealtime support needs’ is not part of the medical file. If the future model consists this or any other specific variable it will consequently be included in the medical file. The variable ‘increasing mealtime support needs’ may for instance be assessed during regular medical screenings by the physician. This way it will become a structural part of the regular care process. The future model will contribute to early identification of markers of FSD. This procedure will help to screen for high‐risk individuals. The model can be seen as complementary to and not as replacement for those screening instruments that already exist. It will help to prioritise high‐risk individuals that should be screened first. By implementing the model, the screening process will be done more automatically and not rely on a single person. Besides it may increase the alertness of the risk for FSD in the organisation.

## 6 Conclusion

FSD is common in adults with intellectual disability and is positively associated with the following variables: increasing mealtime support needs, coughing, cramming food, and having severe–profound intellectual disability. The number of FSD found in this study was 54%.

## 7 Recommendations

In order to develop a prediction model of FSD for adults with intellectual disability based on the associated factors found in the current study, a prospective cohort study is needed. As a follow up of the cross‐sectional data presented here, a longer cohort study is currently ongoing.

In the future, the outcome measure should be objectified by using the gold standard to diagnose FSD in the study population.

Implications for clinical practise suggest gaining more attention in the care organisations for a regular medical screening process for factors associated with FSD, namely increasing mealtime support needs, coughing, cramming food, and having a profound‐severe intellectual disability. In the future, registration of these factors in medical files will help organisations by pre‐selecting a group at risk for FSD that has to be screened first. Besides, special attention should be paid to caregiver training on how to support people with FSD during mealtime.

## Ethics Statement

The study protocol was reviewed by the accredited medical ethics review committee of Erasmus MC Rotterdam (MEC‐2019‐0804).

## Conflicts of Interest

The authors declare no conflicts of interest.

## Data Availability

The data that support the findings of this study are available on request from the corresponding author. The data are not publicly available due to privacy or ethical restrictions.
